# Hospitals in the Smaller Towns1Read at the thirty-fourth annual meeting of the Medical Association of Central New York, held at Buffalo, N. Y., August 27 and 28, 1901.

**Published:** 1901-10

**Authors:** A. L. Beahan

**Affiliations:** Canandaigua, N. Y.


### Hospitals in the Smaller Towns.[1]

By A. L. BEAHAN, M. D., Canandaigua, N. Y.

HOSPITALS where the sick may be cared for are a necessity. In the larger centers of human activity their usefulness has so long been accepted that it would be idle to demonstrate any reason for their establishment. The hospital principle may be embodied in a study of any hospital proposition, whatever the scope of its work. A recent writer states that hospitals in the smaller cities and towns deserve to rank alongside the discovery of anesthetics, as among the most important advancements of medicine and surgery of the past century. No philanthropy nor charity appeals so strongly to our nature as placing within easy reach the means of relieving the sick and wounded sufferers of each and every community. It is an important subject, how to develop the hospitals and adjust them to the needs of smaller communities than those in which they have been established. Should the plan be that of the benevolent and charitable institutions and incorporation be sought under the New York State law now in vogue over half a century, or should some modification of this valuable old working model be adopted? What machinery is required to render available a hospital suited to the needs of

1. Read at the thirty-fourth annual meeting of the Medical Association of Central New York, held at Buffalo, N. Y., August 27 and 28, 1901.

other communities than those now served, in order to extend the work and service in the interests of the individuals to be reached and benefited? The community embraced in the section to reap the reward, and the workers to be fitly recompensed? The private hospital with personal supervision and individual administration has its advantages in direct responsibility, grasp of situation and quick correction of errors.

These hospitals are often the outgrowth of an individual's desire to do better work and are frequently established after experience of service in a public charitable institution. The discussion of preferences between a public and private hospital service for the sick and injured, demands a careful consideration of expediency and adaptability as well as what constitutes high class service and promises ideal results.

A feasible plan for the lesser population of urban communities to meet the needs of all classes would not fit the requirements of a greater population. The different types of patients require different methods of care, but the aim should be to secure equally good results, or else any and all plans and methods of practice are not worthy our emulation or esteem. The work of no hospital should stand unrebuked or unchallenged whose results and whose care of patients can be justly criticised. A standing menace and a disgraceful assumption exist in the classification of patients. Wards should be abolished. Rooms that communicate, with doors between, that nurses may occupy adjoining rooms, and isolation or free ventilation secured, as the case may require, are best. No one patient cared for in an institution is of better clay than another. Wards weaken and make slattern nurses. No nurse should know the price paid by a patient. No room should have a tab with price mark attached. Severe cases should have the best rooms. The less furniture and the best kind for the purposes, gives the cleanest rooms and the most comfortable quarters. The floor should be of hard wood, well seasoned and covered with thin dressings that water and soap if necessary can be used in cleaning and they should be bare and redressed very frequently, making as pleasing an appearance as any fabric can give, and the safest sick-room floor known. The walls should be painted with a washable mixture, and our own preference after a body coat of white lead and oil, is white zinc and turpentine, which makes a clean appearance, is capable of withstanding hardships of cleaning, shows when soiled, and can be reapplied quickly when sepsis has occurred in the room or on that floor.

It is a dull finish and does not tire the eyes. Woodwork and doors are to be of natural wood filled and varnished and revarnished or washed with turpentine and water as often as required.

No drains in the building proper, these being placed with closets and baths; laundry and kitchen in an annex will give an aseptic place for hospital work and if the ground for the building has been properly selected regarding soil and surroundings, we have perfect situation, proper complements and may expect good results. Remodeling of old buildings is not a consistent course. The building should have a southern or eastern exposure in our country, have no filling under or about it and should not be placed among trees whose soil has not been stirred to rid it of the humus and natural noxious principles common to such soil. It should be centrally located in a town, so that during convalescence the activities of the place may give stimulating diversion rather than lonesome isolation and depression. In the work with which I am affiliated, The Beahan Hospital of Canandaigua, N.Y., the village comprises 6,000 inhabitants with contiguous territory of as many additional people. The hospital is incorporated by eight physicians, representing the legally qualified physicians of all the various practices. Each physician and surgeon has control of his own separate work and interests without hindrance. They together constitute its directors and manage its interests. It is a business corporation. It conserves all the interests of local hospital demands without weakening the pecuniary rewards of its members and stands ready with open overtures to care for the indigent and needy as occasion demands when voiced by the charitable instincts and philanthropic purposes of citizens, whose sentiment and hearty coöperation is elicited and encouraged. The project does not interfere with associations whose functions shall be the care of the sick and afflicted poor, but will lead to the formation of such auxiliary bodies.

This result obtains in our town because of advanced and fortunate professional conditions. The new era of medicine, where the best energies of medical and surgical work are directed to the legitimate and scientific study of disease and its remedy, rather than to spend and be spent in professional bickerings and unseemly jangles, arraying communities for or against their medical purveyors, and factions of patrons against each other to the detriment of every one concerned and all their community of interests. No other profession has had such cruel jealousies and such costly strifes. These differences are being abandoned and must be to make the plan of hospital outlined a success.

Not more important that this should be accomplished because of a need of harmony and united effort in the profession, but the community must be taught that esteem for their own physician is best shown by a toleration and respect for other workers in the same profession. To know what it means, and what it costs to develop a hospital work in a smaller town where a surgical center is of necessity to be newly established, reminds one of McDowell's first ovariotomy at Danville, Ky., a town then of only 400 inhabitants. But he did credit to his Virginian teacher and stood ready to face the consequences even of a mob if the operation on Mrs. Crawford proved disastrous. The gratification of better work amply repays for all sacrifices. In our town for the past year no death has occurred from or following a surgical operation in the hospital, nor within ten miles or over a territory of twenty miles square. It may have been a coincidence, it may be a fact that surgical cases met and embraced an opportunity for earlier intervention and better results.

Within the hospital for the last eighteen months section incisions and suture punctures have healed without a drop of pus, even with gauze drains. Hence it is possible that country surroundings contribute to ideal surgery. Establishing new centers for hospital work carries with them the corresponding elevation of standards of nursing and we hope for a class of graduates that will succeed to the places now occupied by the self-asserted nurse of necessity whose unselfish work may be praised, but whose efficiency is below our needs. The difficulty and danger of removing patients to distant cities may be noted, but the procrastination indulged in by the patients invites fatal results and it is probable that the larger centers will reap a benefit in better percentage of recoveries, while the larger experiences of workers in the greater centers of medical and surgical thought will always secure their full share of cases.

The dissemination of hospitals should advance the whole interest of the profession by placing within easy reach of the people a clean, inviting place, where the best care and safest methods constantly checked and corrected may be employed to meet the exigency and direct measures for relief and restoration to health. A large or complex board of managers, comprising persons unacquainted with the problem as a life work, or an establishment with supernumerary assistants to sit on high stools or dawdle away their time, demoralising the work and increasing the cost without bettering returns or records. An organisation with misguided charitable plans that weaken the moral fiber of the indigent or bid for volunteers to fill the beds and but little question of finances asked. A staff of servants who believe themselves dictators, who pull wires to keep themselves in place and corral and control and continue after the hospital interim to care, are all nuisances. Direct work with the least machinery possible in management and work, with personal responsibility in every measure or department with the same high nerve tension of the physician and surgeon, who watches the patient seriously disabled or ill, should permeate the hospital with intense purpose and make it a delicate responsive barometer, whose readings will be met.

The hospitals organised under charitable methods have been ably criticised by Hartwig in his recent retiring address as president of the Buffalo Academy of Medicine. The physician, he says, is imposed upon by this form of hospital. He is a tool, not a manager. He must please the board of control. Free beds are occupied by patients who are able to pay or who are placed there by the influence of large corporations to be paid for by the public. The physicians best appreciate hospital work. Band them together, make their hospitals their centers of professional interests. Their habits of rivalry may be trusted to correct the mistakes of one another.

It does not matter who owns the building or plant. The success of the plant is as dependent on the lesser as the greater stockholder, because in the smaller towns there will never be enough work that a source of material can be alienated with profit to the work and in larger centers, let the physicians' private hospitals conducted on business lines so multiply that their size may not hamper the individual, personal direction of the enterprise.

---

The University of Bellevue Hospital Medical College has a new *x*-ray laboratory which is probably the most complete and practical installation of its kind in this country. It was given to the college as a memorial of the late Edward V. Gibbs, and the donors have provided not only for installing and operating it, but also for keeping it supplied with all the new and improved apparatus that may appear with the development of the science. —*Medical Age.*

